# Knowledge of carbohydrate counting and insulin dose calculations among hospital staff in a regional general paediatrics unit

**DOI:** 10.1186/s40064-015-1469-6

**Published:** 2015-11-24

**Authors:** Jennifer R. O’Gorman, Orla O’Leary, Natalie Finner, Anne Quinn, Clodagh S. O’Gorman

**Affiliations:** The Children’s Ark, University Hospital Limerick, Limerick, Ireland; National Children’s Research Centre, Dublin, Ireland; Centre for Interventions in Infection, Inflammation & Immunity (4i), Graduate Entry Medical School, Limerick, Ireland; Department of Paediatrics, Graduate Entry Medical School, University of Limerick, Limerick, Ireland

## Abstract

The aim of this study was to assess the carbohydrate and insulin knowledge of the staff at Children’s Ark at the University Hospital, Limerick. Carbohydrate counting and insulin dose calculations based on carbohydrates and blood sugars are integral to intensive insulin management of type 1 diabetes mellitus (T1DM). The PedCarbQuiz, a validated questionnaire, was modified, and applied to the staff on our general paediatrics ward. 48/70 eligible staff responded (rate 68 %). Overall knowledge was good: 75.5 % was the average score for correctly identifying foods containing carbohydrate. However, poor scores were obtained for calculating multiple items and meal values (average score 29 %), and exact values of insulin required (average score 38 %). These results highlight the need for re-education among staff on a general paediatrics ward, to empower ward staff to contribute effectively to the education and management of patients with T1DM.

## Background

The intensive management of Type 1 diabetes mellitus (T1DM) in children requires insulin regimes that are dose adjusted based on the carbohydrate content of a meal and the patient’s blood glucose (Silverstein et al. 2005). Implementing this requires knowledge about the carbohydrate content of foods, and subsequently the ability to calculate the correct insulin dose. This a fundamental task carried out several times daily by patients with T1DM who are on intensive insulin regimes. Paediatric patients and their families are taught the principles of this during T1DM education and re-education sessions by their T1DM multi-disciplinary team (MDT).

The implications of poor glycaemic control are well known. Good control in children is imperative to maintain normal physical growth (Silverstein et al. 2005), to avoid acute complications of ketoacidosis and hypoglycaemia, as well as combating against chronic microvascular and macrovascular complications (Nathan 2014; Brink 2001; Olsen et al. 2000). The unique challenges faced when managing T1DM in children are also recognised in the literature, including increasing diagnoses in younger children (Edwards 2014; Streisand and Monaghan 2014), evolving needs as patients grow and develop, and continual support and education required across different environments including at home and at school (Edwards 2014; Borus and Laffel 2010).

Hospital admissions are an excellent an opportunity to identify poor control and reinforce carbohydrate and insulin principles to patients and parents, if the staff at ward level are competent in these principles. The aim of this study was to assess the level of knowledge of these carbohydrate and insulin calculations by paediatric ward staff members who are not part of the T1DM MDT, and thereby identify how well we are using this opportunities to re-educate our patients.

## Methods

We assessed the knowledge of the ward staff at The Children’s Ark, University Hospital, Limerick (UHL), regarding carbohydrate content of food, carbohydrate counting and insulin dose calculations using the validated PedCarbQuiz (PCQ) questionnaire. The PCQ was designed and tested in the Department of Paediatrics, Rainbow Babies and Children’s Hospital, Case Western Reserve University, Cleveland, Ohio, a tertiary paediatric diabetes clinic (Koontz et al. 2010). While it is designed for a US paediatric diabetes cohort, we have modified it for an Irish population and used it in a previous study in an Irish paediatric diabetes cohort (submitted data). The questionnaire evaluated the staff members’ understanding of the carbohydrate content of commonly eaten foods, the ability to read nutritional labels, and the calculation of proper insulin dosage. A report is generated which estimates skills in three domains: calculating carbohydrate content; insulin dose calculations; and overall skills (Koontz et al. 2010).

Modification of this questionnaire for an Irish population included: (1). Replacing certain typically American or Canadian food groups with more typical Irish foods, e.g. corn dogs with sausages; and (2). Conversion of the blood sugar readings within the PCQ from American (mg/dL) to Irish units (mmol/L). The PCQ was then offered to 70 members of paediatric ward staff (45 nurses and 25 doctors) who were rostered to work over a 2 weeks period. Questionnaires were filled out and returned anonymously to a collection box located on the ward. The grade of staff was highlighted on returned studies, but no other identifiable information was collected. Members of the T1DM MDT were excluded from participating in this study.

Results were calculated using the marking scheme devised in the original questionnaire. The survey contained seven sections. These included carbohydrate recognition, carbohydrate counting of individual items, carbohydrate calculation of an entire meal, nutritional label reading, use of an insulin sliding scale, use of insulin to carbohydrate ratios, and calculation of whole meal insulin dose using all of the above knowledge domains.

Local institutional ethical approval for this study was granted.

## Results

48 out of 70 eligible staff responded (rate 68 %), including 34/45 (76 %) nurses and 14/25 (56 %) doctors. The overall knowledge of staff was good. The average mark obtained for correctly identifying foods containing carbohydrate was 75.5 %, and the majority of staff members scored highly at nutritional label reading (average department total score 94 %), use of insulin sliding scale (average department total score 90 %) and use of insulin carbohydrate ratios (average department total score 94 %).

However, scores were lower for the ability to count the amount of carbohydrates in individual items (average department score 29 %), and the amount of carbohydrates per meal (average department score 26 %). Carbohydrate counting for a meal is an integral requirement for calculating insulin doses, and thus this led to poor scores for calculating insulin doses for meals (average department score 38 %).

All staff members achieved a higher score in the insulin dosing domain compared to the carbohydrate counting domain. Consultants demonstrated a greater amount of knowledge on average in comparison to the other staff members. [See Tables 1 and 2 for summaries of scores for nurses (registered general nurses and clinical nurse managers), and doctors (non-consultant hospital doctors and consultants) separately.]

**Table 1:** Summary of scores in each domain according to staff grade

	Registered general nurse (RGN) (N = 30)	Clinical nurse manager (CNM) (N = 4)	Consultant doctors (N = 4)	Non-Consultant hospital doctors (NCHD) (N = 10)	Department total (N = 48)
Carbohydrate recognition (%)	69	74	85	74	75.5
Carbohydrate counting (individual item) (%)	35	21	41	18	29
Carbohydrate counting (entire meal) (%)	29	38	23	14	26
Nutritional label reading (%)	92	96	100	88	94
Use of insulin sliding scale (%)	85	89	98	88	90
Use of insulin to carbohydrate ratios (%)	95	100	100	80	94
Calculation of whole meal insulin dose (%)	43	30	45	35	38

**Table 2:** Summary of average total score, average carbohydrate score and average insulin score according to staff grade

	Average total score (%)	Average carbohydrate score (%)	Average insulin score (%)
Registered general nurse (RGN) (N = 30)	69	55	74
Clinical nurse manager (CNM) (N = 4)	66	57	73
Consultant doctor (N = 4)	74	62	87
Non-consultant hospital doctor (NCHD) (N = 10)	65	49	67
Total	68.5	55.7	75.2

## Discussion

Our study demonstrated greater knowledge among all staff members of insulin dosing in comparison to carbohydrate counting. This is consistent with current ward practice, with patients estimating the carbohydrate content of meals, and the nursing and medical staff double-checking the patient’s calculation of insulin required based this estimate.

The average total score for the department as a whole was 68.5 %. In another study using the PCQ questionnaire (unpublished), patients with T1DM attending our paediatric clinic had an average total score of 68.9 % ± 15.8, which is comparable to the score for staff in this study. The total average patient carbohydrate score was 68.7 % ± 16.3 versus 55.7 % among staff members. This highlights the discrepancies in our knowledge to advise our patient’s on their diet, and on carbohydrate intake. Furthermore the total average PCQ score in the original American study was 87.9 % ± 9.7 (Koontz et al. 2010). Our total average score is significantly less than this. Of note all patients in the American study were taught carbohydrate and insulin skills by a dietician. There were no dieticians included in our study group, and at the time this study was conducted, there was no dietitian for paediatric diabetes patients working in our unit. It is possible that staff knowledge of carbohydrates would improve if a dietitian was working on the ward, providing dedicated education to children and families but also some education, either formal or informal, to other staff members. Despite this, it cannot be ignored that staff knowledge is lacking, and that we may be missing valuable opportunities to reinforce carbohydrate counting techniques during inpatient stays.

Overall, consultant doctors scored the highest, followed by RGNs and CNMs, with non-consultant hospital doctors scoring the lowest. While it is important that all staff have knowledge of carbohydrate content of foods, it is arguably a more important skill for hospital nurses than for hospital doctors, as it is usually nurses who have more direct patient contact during hospital admissions. Notwithstanding, it is clear from the results of this study, that staff on our general paediatrics ward are not equipped to provide constructive ongoing help or education to patients when trying to count carbohydrates and then calculate relevant insulin doses. It is not likely that every staff member could aim to be an expert carbohydrate counter but it is possible to aim to improve everyone’s baseline level of knowledge and to aim to have some staff members who are experts in this domain, who might then act as champions for carbohydrate counting and insulin dosing accurately.

One of the limitations of this study is that PCQ was developed for a US population—some food types included in the original validated questionnaire are not consumed regularly in Ireland. We modified the questionnaire to better suit our study population, and although it has been used in a previous paediatric study in the department, it has not been fully validated. A further limitation is the response rate to the study. A response of 68 % does not reflect knowledge of department as a whole. For re-auditing in the future, we will emphasise that each questionnaire is anonymised, and the importance of the results for improved clinical practice on the ward.

This study has highlighted the need for education of staff members in the department, particularly with regards to exact carbohydrate content of food. This may be achievable following the appointment of a specialist paediatric diabetes dietitian. We propose education in the format of departmental lectures, a practical tutorial session with emphasis on carbohydrate counting and subsequent insulin dosing, and ongoing awareness and education with picture guides as aide-memoires on the wards. A further option would be for the catering service to standardise carbohydrate content of food served to children with T1DM during in-patient admissions.

## Conclusion

The key finding from our study was a significant discrepancy in staff members’ knowledge of carbohydrate counting and insulin-dosing in T1DM on a general paediatric regional hospital ward. This will have a direct impact on the management of paediatric patients under the department’s care. We are well aware of the long term implications of poor diabetic control, and the particular challenges facing management of T1DM in children, and yet we are not utilising inpatient hospital admissions as an opportunity for re-education for the patient with T1DM. Staff education is required, as well as stronger paediatric dietetic presence. Following re-education of staff members we propose repeating this study.
